# Genetic Markers of the Host in Persons Living with HTLV-1, HIV and HCV Infections

**DOI:** 10.3390/v8020038

**Published:** 2016-02-03

**Authors:** Tatiane Assone, Arthur Paiva, Luiz Augusto M. Fonseca, Jorge Casseb

**Affiliations:** 1 Laboratory of Dermatology and Immune deficiencies, Department of Dermatology, University of São Paulo Medical School, LIM56, Av. Dr. Eneas de Carvalho Aguiar 500, 3rd Floor, Building II, São Paulo, SP, Brazil; 2 Institute of Tropical Medicine of São Paulo, São Paulo, Brazil; arthurmpaiva@usp.br; 3 Department of Preventive Medicine, University of São Paulo Medical School, São Paulo, Brazil; augustom@hcnet.usp.br

**Keywords:** HTLV-1, HIV-1, HCV, genetic factors

## Abstract

Human T-cell leukemia virus type 1 (HTLV-1), hepatitis C virus (HCV) and human immunodeficiency virus type 1 (HIV-1) are prevalent worldwide, and share similar means of transmission. These infections may influence each other in evolution and outcome, including cancer or immunodeficiency. Many studies have reported the influence of genetic markers on the host immune response against different persistent viral infections, such as HTLV-1 infection, pointing to the importance of the individual genetic background on their outcomes. However, despite recent advances on the knowledge of the pathogenesis of HTLV-1 infection, gaps in the understanding of the role of the individual genetic background on the progress to disease clinically manifested still remain. In this scenario, much less is known regarding the influence of genetic factors in the context of dual or triple infections or their influence on the underlying mechanisms that lead to outcomes that differ from those observed in monoinfection. This review describes the main factors involved in the virus–host balance, especially for some particular human leukocyte antigen (HLA) haplotypes, and other important genetic markers in the development of HTLV-1-associated myelopathy/tropical spastic paraparesis (HAM/TSP) and other persistent viruses, such as HIV and HCV.

## 1. Introduction

Human T-cell leukemia virus type 1 (HTLV-1), hepatitis C virus (HCV) and human immunodeficiency virus type 1 (HIV-1) have been reviewed, since these viruses are prevalent worldwide, and share similar means of transmission, and superposition of infected populations, such as intravenous drug users (IDU) and commercial sex workers, especially in endemic areas for HTLVs. In addition, these infections may influence each other in evolution and outcome, including cancer or immunodeficiency.

Many studies have reported the influence of genetic markers on the host immune response against different persistent viral infections, such as HTLV-1 infection, pointing to the importance of the individual genetic background on their outcomes. However, despite recent advances on the knowledge of the pathogenesis of HTLV-1 infection, gaps in the understanding of the role of the individual genetic background on the progress to clinically manifested disease still remain. In this scenario, much less is known about the influence of genetic factors in the context of dual or triple infections, or their influence on the underlying mechanisms that lead to outcomes that differ from those observed in monoinfection. HTLV-1 is an ancient infection, with better adaptation to its host than HIV or HCV. HIV often complicates the evolution of HCV and HTLV-1, but the reverse may not be true, with a higher spontaneous clearance rate of HCV in those triple co-infected with HCV/HIV/HTLV than in those with HCV/HIV, or monoinfected with HCV.

This review describes the main factors involved in the virus–host balance, especially for some particular human leukocyte antigen(HLA) haplotypes, and other important genetic markers in the development of HTLV-1-associated myelopathy/tropical spastic paraparesis (HAM/TSP) and other persistent viruses, such as HIV and HCV.

## 2. HLA Risk for HAM/TSP

HAM/TSP occurs in only 1%–2% of those infected by HTLV-1,thus, host genetic factors interacting with viral factors have been suggested as determinant to the outcome; that is, whether the individual will develop an effective immune response to HTLV-1 or will progress to clinically manifested HAM/TSP. Among those genetic factors, the most frequently studied have been the haplotypes of HLA Class 1 (HLA-A, HLA-B, HLA-C) of the major histocompatibility complex (MHC), molecules that encode glycoproteins expressed on the surface of almost all nucleated cells, and of which the major function is the presentation of antigenic peptides to CD8+ T lymphocytes. The effectiveness of a specific immune response to HTLV-1, especially the cytotoxic CD8+ T lymphocytes’ response (CTL), has been shown to be the key to control the provirus load (PVL) of HTLV-1 [1]. The most compelling evidence for a role of host CTLs came from the observation of a population in Southern Japan, where the presence of two genes of the HLA class 1 (HLA-A*02 or HLA-Cw*08) was associated with a lower PVL and decreased prevalence of HAM/TSP [2,3].

Some specific HLA alleles have been associated with protection, while others have been associated with an increased risk of HAM/TSP. However, unlike the expressions of both HLA-A*02 and HLA-Cw*08, which are associated with a protective effect, HLA-DRB1*0101 and HLA-B*5401 have been linked with an increased susceptibility to HAM/TSP [2,3]. The association of HLA-DRB1*0101 with disease susceptibility only becomes evident in the absence of the HLA-A*02 protective effect [2], whereas HLA-B*5401 is independently associated with susceptibility to disease; moreover, among patients with HAM/TSP, HLA-B*5401 is associated with a significant increase in PVL. HLA-A*02, HLA-Cw*08 and *HLA-DR1* are also found in the population of Southern Japan, where they are associated with a higher risk for HAM/TSP [4].

There may be differences in the frequency of HLA alleles in different populations, and changes in the protective effect of certain HLA alleles according to ethnicity (Table 1). The same protective effect of HLA-A*02 in HAM/TSP, seen in Japanese, has been reported in a small sample of 29 individuals from London, 27 of whom had a Caribbean origin [2], a finding also observed in Brazil [5,6] but not in other populations, such as Afro-Caribbean individuals from Martinique [7], Jamaica [8], Spain [9] and Iran [10,11].

HTLV-1 PVL is controlled by the host immune response, with a dominant role for an effective CTL response [8]. Long-term studies have shown that CTL is determinant of the outcome; for example, in Japanese cohorts, HLA-A*02 and HLA-C*08 play a protective role against the development of HAM/TSP, whereas HLA-B*54 is associated with a higher risk. This outcome is possibly related to the killing of infected cells with *HLA-A2* or -C*08 restricted HTLV-1 epitopes, resulting in decreased PVLs [9]. In fact, such HLAs have the ability to present peptides derived from viral proteins to CD8+ T cells, which are mostly protective during HTLV-1 infection [10].

**Table 1:** Distribution of human leukocyte antigen (HLA) haplotypes according to risk of HTLV-1-associated myelopathy/tropical spastic paraparesis (HAM/TSP) development.

HLA Allele	Japanese	Brazilians	Iranians	Spanish	Afro-Caribbean (Martinique)	Afro-Caribbean (London)	Jamaicans
A*02	++	+	0	0	0	++	0
Cw*08	++	0	0	0			
A*24	-		0		0		
B*07	-	±	0	-	0		0
B*5401	-	ᴓ	ᴓ	ᴓ	ᴓ	ᴓ	ᴓ
DRB1*0101	±	0	±	-	0		0
DRB1*11	-	-	-		-	0	0

++ protective effect; + tendency to protective effect; - susceptibility; ± susceptibility only in negative HLA-A*02; 0 no associated effect; ᴓ HLA not prevalent.

In Iran, alleles HLA-A*02, HLA-Cw*08 and HLA-A*24 were not associated with a lower risk of HAM/TSP or lower provirus load [10,12]. In Brazil, HLA-Cw*08 showed no protective effect, and, among HLA-B*07 individuals, only those negative for HLA-A*02 [6] were susceptible to HAM/TSP [6]. In Spain, no association between the presence of protective alleles (HLA-A*02 and/or *HLA-Cw08*) and HAM/TSP could be demonstrated nor were there significant differences in PVLs; however, HAM/TSP was significantly associated with HLA-B*07 and HLA-DRB1*0101 [9]. The HLA-B*5401 allele was not found in the populations of Iran, Brazil and Spain (Table 1), and has been described almost exclusively in East Asian individuals [13].

Alleles associated with a higher risk, such as HLA-DRB1*0101, were also associated with susceptibility to HAM/TSP in Iran’s population, as well as in Japanese, an effect was observed only among HLA-A*02-negative individuals, and not occurring in HLA-A*02-positive individuals [10]. The association of HLA-DR*11 with HAM/TSP, previously described in Japanese patients, was observed only in Brazilian patients [5]. Among Brazilian individuals, HLA-Cw*07 was associated with HAM/TSP only in the absence of HLA-A*02 [6].

The protective ability of HLA class 1 allele correlates with the affinity to bind antigenic peptides derived from the HTLV-1 proteins [14]. However, contrary to what was expected, HTLV-1 antigen, which is recognized by the protective immune response class 1 immune dominant Tax, was not associated with this protein, but rather with the regulatory protein encoded hemoglobin subunit zeta (HBZ) on the negative strand of the provirus. A combination of theoretical methods for the prediction of epitopes [15] and cellular laboratory experiments demonstrated that the binding to epitopes of the protective HLA-A*02 and HLA-Cw*08 alleles are stronger than that of the detrimental HLA-B*54 [14]. In that study, HLA class 1 molecules that bind strongly to HBZ epitopes were significantly associated with the asymptomatic state, an association remaining even after patients with HLA-A*02, HLA-A*08 and HLA-B*54 were excluded from the analysis, demonstrating that the protective effect of binding HBZ is common to several HLA alleles and not just a feature of particular alleles. Moreover, among both asymptomatic subjects and HAM/TSP patients who carry protective alleles, epitopes that could bind HBZ were strongly associated with a significant reduction in HTLV-1 PVL [14].

## 3. Interferon Lambda 3 (IFN-λ3)

IFN lambda 3 (IFN-λ3) is an important cytokine that is responsible for an unspecific antiviral response by interacting with the HLA class II receptor, inducing intracellular signaling by janus kinase/signal transducers and activators of transcription (JAK/STAT) and mitogen-activated protein kinases (MAPK). Host genetic background in HLA class II, encoded by single nucleotide polymorphisms (SNPs), can lead to a spatial conformation in the receptor, modifying the attachment that avoids interaction between IFN-λ3 and its receptor, inducing a genetic by stand interaction [16].

The first reports about the role of *IL28B* (coding for IFN-λ3) on HAM/TSP outcomes could not clearly show the connection [17,18]. However, it was noted that HAM/TSP patients presented an independent association with the polymorphism in *IL28B* SNP rs8099917 (GG), when compared to asymptomatic HTLV-1 carriers [19]; such a finding has not been reported for other infections, such as HIV and HBV infections [20], except for patients with acute HIV infection, whose response to antiretrovirals was related to SNP rs12979860 [21].

In recent years, an association between IFN-λ3 polymorphisms and anti-HCV treatment with pegylated interferon (PEG-IFN) outcome was described [22]. The correlation between the polymorphism of IFN-λ3 was noted in two positions (rs12979860 and rs8099917) [16,22]. Co-infection with HIV or HTLV is widely present among HCV subjects, making interaction a possibility, and potentially changing both the pathogenesis of the disease and/or the response to treatment [23,24].

It is noteworthy that the immune response seems to be a crucial factor in the pathogenesis of HAM/TSP. For example, a study showed that patients with HAM/TSP had higher levels of IFN-gamma compared to asymptomatic patients [25]. Furthermore, the polymorphism of rs12979860 SNP profile induces the production of IFN-λ3 and an immune response to HTLV-1, leading to neuronal injury in the spinal cord [26,27]. As reported, the interferon stimulated genes likely regulate the expression of cytokines and this regulation may differ in the infected tissue and between cell types within the liver and spinal cord [28]. It is known that IFN-λ3 attenuates interleukin (IL)-13 production, leading to a protective effect and decreased inhibition between killer-cell immunoglobulin-like receptor (*KIR*) and *HLA-C* [29]. Thus, it is possible to infer that IFN-λ3 and two other interferons, IL-28A and IL-29, can activate the JAK-STAT cascade, which is similar and probably synergistic with type 1 interferons (e.g., interferon alpha), although using different receptors, and contributing to the immune pathogenesis of HAM/TSP.

## 4. miRNA

The importance of microRNA (miRNA) in the replicative cycle of several other viruses, as well as to the progression of associated pathologies, was established over the past decade. Furthermore, the involvement of miRNAs in altering the life cycle of HTLV-1 and progression to neurodegenerative diseases and related oncogene has been investigated [30]. Various miRNA-derived transcript proteins can change the features of HTLV-1, either interacting with the restructuring of chromatin or manipulating components of the RNA interference (RNAi), by providing multiple routes through which miRNA expression, *etc.*, can be down-regulated in the cell host. Furthermore, the mechanism of action by which deregulation of host miRNAs can affect cells infected with HTLV-1 can vary substantially through the silencer, including miRNA-induced silencing complex of RNA (RISC), gene transcription, inhibition of components RNAi, and chromatin remodeling. These changes induced by miRNA can lead to increased cell survival, invasiveness, proliferation and differentiation; they can also allow viral latency. Recent studies have shown the involvement of successful miRNAs in the life cycle and pathogenesis of HTLV-1, but there are still significant issues to be addressed. In Table 2, a summary of the already identified miRNAs and their biological effects on HTLV-1 are presented. In HAM/TSP, the miRNA involved in the pathogenesis is the miR-132, while, in adult T-cell leukemia/lymphoma (ATL), miR-223 is responsible for promoting oncogenesis, an important biological marker [31].

HTLV-1 can change the miRNA profiles of infected cells, contributing to cell transformation and leading to the development of ATL and/or HAM/TSP [30]. Moreover, the modification of chromatin by viral proteins and host cell miRNAs may contribute to the deregulation of cells expressing miRNA and may possibly serve as a key mechanism by which the virus manipulates the status of miRNA host. Recent discoveries attempt to validate the importance of variation in the levels of miRNA mediated by HTLV-1, but there is a gap on this recent field of study. Moreover, a better understanding of the molecular mechanisms may contribute to a better understanding of viral regulation and cellular regulatory pathways. Taken together, this knowledge can identify potential therapeutic intervention points in the future [31].

**Table 2:** Identified miRNAs and their biological effects on HTLV-1.

MiRNA	Regulation	miRNA Target	Function
miR-21	Upregulated	PTEN	Antiapoptotic
miR-93	Upregulated	p21 (WAF1/CIP1); MICB	Antiapoptotic
miR-132	Downregulated	p300	Immune evasion
miR-143-p3	Upregulated	AChE; PKA; GRα	Increase of viral transcription
miR-146 a	Upregulated	Unknown	Pro-inflammatory
miR-149	Downregulated	p300	Proliferation
miR-155	Upregulated	TP53INP1; Unknown	Proliferation
miR-873	Downregulated	p300	Proliferation

PTEN: Phosphatase and tensin homolog; MICB: MHC class I polypeptide-related sequence B; AChE: *Acetylcholinesterase*; PKA: Protein kinase A; GRα: glucocorticoid receptor α; TP53INP1: Tumor protein P53 inducible nuclear protein 1.

## 5. Killer Cell Immunoglobulin-Like Receptors (KIRs)

KIR present a high tendency to suffer genetic mutations, indicating a high polymorphic capacity. HLA-C molecules present ligands for KIR2DL receptors, with a functionally relevant indistinct to determine KIR specificity, like *HLA-C* group 1 (*HLA-C1*) alleles, where the HLA-C alpha 1 domain is ligand for the inhibitory receptors KIR2DL2 and KIR2DL3 and the activating receptor KIR2DS2 [32,33]. *HLA-C* group 2 (*HLA-C2*) alleles are involved in inhibitory KIR2DL1 and in the activation of KIR2DS1 [32,33,34,35]. KIR2DL3 and its ligand HLA-C1 have been associated with an increased likelihood of spontaneous [36,37] and treatment-induced HCV clearance [37,38]. This association is attributed to differential natural killer (NK) cell activation and function in the context of this KIR/HLA interaction [39]. SNPs from the *HLA-C* coding regions show weak associations with sustained virologic response(SVR) [16,40] for chronic hepatitis C.

**Figure 1:**
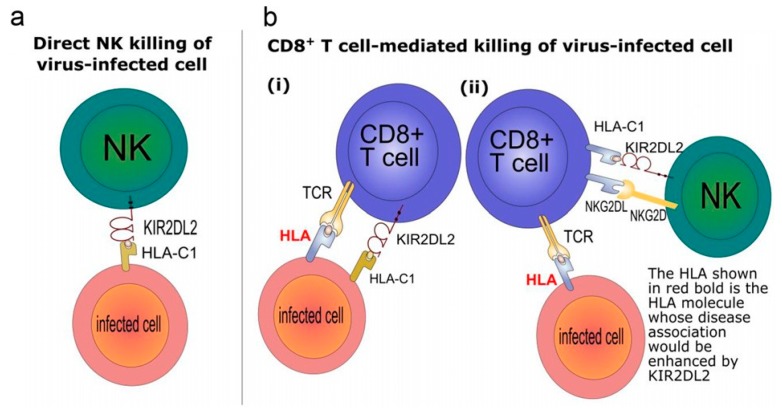
Mechanisms explaining inhibitory killer-cell immunoglobulin-like receptor (KIR) enhancement of human leukocyte antigen (HLA) class I associations.

New evidence has shown that the pathogenic mechanism of disease-associated HTLV-1 infection is an impairment of the immunity [41]. The KIR genotype influences CTL efficiency, by affecting HLA class I-mediated HTLV-1 immunity [42], and KIRs influence both innate and adaptive immunity (Figure 1) [1,42], however KIR2DL2 gene is associated with an enhancement of the effect of known protective or detrimental HLA class I alleles on PVL and HAM/TSP risk, for multiple HLA-A, -B and -C molecules. Surprisingly, KIR2DL2 also exhibits the same behavior in HCV infection, another unrelated virus. In HTLV-1 infection, KIR2DL2 enhanced the protective and detrimental effects of HLA-C*08 and B*54, respectively, on disease status and enhanced the association between B*54 and high PVL in HAM/TSP patients [42]. In HCV infection, KIR2DL2 enhanced the protective effect of B*57 on its spontaneous clearance and the association between B*57 and low viral load in chronic carriers [42]. These observations suggest that KIR2DL2 enhancement of the HLA class I-restricted response may be a general mechanism.

## 6. Genes and Susceptibility to HIV Infection

Susceptibility to HIV infection and the clinical course after infection are both influenced by the complex interaction of factors related to the human host, the virus and the surrounding environment, resulting in large epidemiological and clinical heterogeneity among infected individuals. Host genetic factors play an important role in this variability and pathogenesis. For example, the gene encoding the CCR5 (chemokine (C-C motif) receptor 5) co-receptor necessary for infection of R5 strains of HIV, which usually initiate infection, and may influence both the acquisition of infection and the rate of progression to disease.

The *CCR5* gene is located on chromosome 3 and individuals with *CCR5 Δ32* deletion acquire protection against virus to produce a defective protein, which is not expressed on the cell surface, preventing the virus from binding to the CCR5 co-receptor to penetrate the cell. While homozygous for *CCR5* deletion has Δ32-protection against HIV [43,44,45], heterozygous for the mutation has a delay of two to four years of progression to acquired immune deficiency syndrome (AIDS) [46,47,48]. However, homozygous for the *CCR5Δ32* deletion mutation is the only genotype identified as being capable of protecting against HIV infection. The *CCR5Δ32* allele occurs at a frequency of 4% to 15% in the Caucasian population, with a higher frequency in the Northern European populations [44,49]. Other genetic potential protective effects against infection caused by deletions seem to involve more complex interactions between two or more gene variants. Apparently, Asians and Africans with *CCL5-403A/A* genotype (chemotactic chemokine ligand 5), also called RANTES (regulated on activation, normal, T cell expressed and secreted), could be resistant to HIV-1 but controlled studies are still lacking to confirm this observation [50]. In China, a genetic variant of SDF-1, the primary ligand of CXCR4 ((C-X-C motif) receptor 4), was associated with resistance to HIV infection in intravenous drug users [51].

ZNRD1 (zinc ribbon domain-containing protein 1) is a DNA-dependent RNA polymerase catalyzing the transcription of DNA into RNA required to complete the life cycle of HIV, which was subsequently identified as SNPs associated with depletion of CD4+ T cells. A haplotype in ZNRD1 gene was associated with a 35% reduction in the risk of HIV acquisition in Euro-Americans (Americans with European ancestry) and ZNRD1 variants also affect the progression of HIV-1 infection to disease in European, American, and African cohorts [52].

Langerin, also known as CD207, is a transmembrane receptor encoded by the *CD207* gene and expressed in Langerhans cells, scattered throughout the genital mucosal epithelium where the transmission of HIV occurs. Although Langerhans cells are considered dendritic cells (DC), and immature DCs are involved in the transmission of HIV to lymphocyte T [53], there is evidence suggesting that Langerin prevents HIV transmission. The HIV particles captured by Langerin are internalized and degraded in the Birbeck granules [54]. However, a mutation in the gene of persons deficient in the Langerin Birbeck granules has been described [55].

Defensins are small cationic proteins rich in cysteine produced by leukocytes and epithelial cells that are active against bacteria, viruses, and fungi. They play a role in immunity penetration through the cell membrane and pore formation flue material [56,57,58]. Mammalian defensins are classified as alpha, beta, and theta defensins [58]. Alpha-defensin bound to receptor CD4 and gp120 glycoprotein of the viral envelope may negatively modulate CD4+ T cells and inactivate viral particles through disruption of membrane [59,60]. Accordingly, they could block HIV entry directly by inactivating it or by blocking or eliminating viral receptor on the cell surface. Beta-defensins have mechanism of action similar to that of alpha-defensin, blocking virus entry of both the tropic virus strains macrophage (R5 viruses), as well as tropic strains for T cells (X4 viruses), achieving its effect by direct inactivation of viral particles or negative modulation of CXCR420 [61]. Six human beta-defensins were identified in epithelial cells, although they can be present in up to 28 different human genes [62]. Theta-defensins in humans and chimpanzees are only found as inactive pseudogenes, which are transcribed into mRNA, but are home to premature stop codons that prevent expression of functional products [63]. However, by reconstituting the human putative ancestral gene for theta-defensin, the presence of potent activity against strains X422 and R5 was observed *in vitro*. The reconstituted product, called human retrociclin-1, binds to CD4 molecule and gp120, preventing viral entry into target cells [64].

TREX-1 (three prime repair exonuclease), which degrades cytosolic DNA, preventing unnecessary immune response against free nucleic acids, is a limiting factor for HIV-1 and polymorphism of a single nucleotide rs3135945 and was associated with susceptibility to HIV infection, emphasizing the participation of TREX-1 in anti-HIV response [65].

### 6.1. Genes that Influence the Dynamic Progression of AIDS

Apart from individuals uninfected by HIV, despite repeated sexual exposure to the virus in high risk situations, known as exposed uninfected, there is a small proportion of HIV-infected individuals who remain clinically and immunologically healthy for more than one or two decades after being seroconverted, while in others infection may be characterized by an extremely rapid progression to AIDS within one year [66]. Host genetic factors possibly contribute to this heterogeneity, as demonstrated by many studies in which genetic polymorphisms in human genes are able to influence the risk of HIV infection and progression to AIDS [66].

The type of HLA is one of the host genetic factors associated with the course of HIV infection. The *HLA-B* alleles are considered the primary genetic determinants of disease progression, according to the categorization in rapid progressors, slow progressors and long-term non-progressors [67]. While *HLA-B35* is associated with rapid progression to AIDS [67,68,69], HLA-B*5701 and *HLA-B27* are more prevalent among long-term non-progressors (LTNP) [70,71,72,73,74,75], of which 1% are the elite controllers (EC), who are mainly characterized by maintaining persistently undetectable viral loads without antiretroviral treatment.

Associations among the SNPs of MHC class I, MHC class III and LTNP phenotype are observed [76] is also noted in other factors, such as the co-expression of multiple HLA protectors, SNPs of HLA-C, and stronger T cell responses against the HIV proteins in elite controllers individuals [77]. These different genetic variant combinations may have addictive, synergistic or inhibitory effects determining the course of HIV infection. HLA class II also contributes to the immune response in the control of the viral load of HIV patients and distinct stratifications of *HLA-DRB1* effect on HIV viremia between controllers and progressors are associated with different subsets of HLA-DRB1 alleles, with DRB1*15:02 significantly associated with low viremia and DRB1*03:01 with high viremia [78,79].

Unlike individuals with two copies of the mutation *CCR5Δ32* who are protected from HIV infection by non-functionality of *CCR5* [43,44,45], heterozygous individuals for this mutation can be infected by HIV R5 strains but exhibit an altered activity of the chemokine receptor, resulting in delayed progression to AIDS [46,47,48]. Occurring at a frequency of up to 15% in the Caucasian population, especially in the Northern European population, the allele *CCR5Δ32* is virtually absent among natives of Africa and its marginal presence in Asian populations may be due to gene flow from Caucasian populations [80,81,82].

Polymorphism in otherwise apparently normal chemokine receptors also has some degree of influence on disease progression. For example, CCR2 chemokine receptor can function as a HIV co-receptor in some situations [83]. Person with homozygous or heterozygous for *CCR2-64I* which results mutation from valine to isoleucine changed at amino acid position 64, progress more slowly to AIDS than those homozygous for the wild type variant, although not all studies confirm this association [84]. There is also controversy as to CX3CR1, fractalkine, a receptor for chemokine, for which the initial studies showed an association with more rapid progression to AIDS [85,86,87].

The beta-chemokines MIP-1α (CCL3), MIP-1β (CCL4) and RANTES (CCL5) are natural ligands of CCR543. Two natural variants have been described and named CCL3L1 and CCL4L147. CCL3L1 (also known as MIP-1αP) is the most potent CCR5 agonist and is a strong inhibitor of infection by R5 strains of HIV-1 [88,89]. RANTES (CCL5), through promoters 28G-403A, could also slow progression to AIDS, whereas RANTES has another variant, named 1.1.C, which accelerates AIDS development [84].

The LTNP condition probably results from a complex association of various genetic factors rather than only one variant of a single gene, as confirmed by the later discovery of two new association between allelic variants of *TNF-a-238* genes and *PDCD1-7209* and LTNP situation [90].

### 6.2. Genes Important to Anti-HIV Treatment

Abacavir is a reverse transcriptase inhibitor antiretroviral used in current clinical practice with other antiretroviral agents with few interactions with other drugs and favorable long-term toxicity profile. However, it has the most important adverse effect on the immune-mediated hypersensitivity reaction, affecting 5% to 8% of patients in the first weeks of treatment, requiring the immediate cessation of treatment [91]. Their subsequent reintroduction is contraindicated due to the risk of recurrence of the reaction with greater severity, speed and risk of death [91]. A hypersensitivity reaction to abacavir is strongly related to the presence of the HLA-B*5701 [92,93,94,95,96] and avoiding abacavir in patients with HLA-B*5701 reduces the incidence of that reaction [97]. The effectiveness of screening for HLA-B*5701 in preventing hypersensitivity reaction to abacavir has been established, although its cost-effectiveness depends on factors that vary between populations and health care settings, and the availability of test [98].

The observation that individuals homozygous for the *CCR5-Δ32* deletion show protection against HIV led to the development of drugs antagonist of the CCR5 chemokine receptor, blocking this receptor and inhibiting HIV entry that uses CCR5. As the CCR5 antagonists administration carries the risk of selection of viral variants able to use alternative CXCR4 co-receptor, tropism for co-receptor should be assessed prior to clinical use of inhibitor [99]. Maraviroc was the first antiretroviral drug with this mechanism of action; other drugs of this group include vicriviroc, cenicriviroc, adaptavir, INCB-9471 and PRO-140.

In 2007, an HIV-infected adult patient living in Berlin developed acute myelogenous leukemia and was treated with a transplant from an allogeneic hematopoietic stem cell donor who was homozygous for the *CCR5-Δ32* deletion and, after stopping antiretroviral therapy following transplantation, his viral load remained undetectable, becoming the first confirmed case of cure of HIV infection [100]. Since then, several efforts are underway in an attempt to reproduce the Berlin patient’s condition through the engineering of autologous T cells or hematopoietic stem cells resistant to HIV [100].

### 6.3. Co-Infection HIV–HTLV

HTLV-1 and -2 have the same modes of transmission as HIV, resulting in common risk factors and overlapping of populations exposed, so that in individuals infected with HIV, HTLV is 100 to 500 times more frequent than in the general population. *In vitro*, *Tax* gene products of HTLV-1 increment the release of free viral particles of HIV-1 [101] and an accelerated course of HIV-1 infection has been reported in patients co-infected with HIV-1 and HTLV-1 [24,102,103].

On the other hand, the Tax protein of HTLV-2 may have an immunomodulatory effect, increasing IFN-y synthesis by cells infected with HIV-1 [104], and HTLV-2 induces the production of CCL3 chemokine, CCL4 and CCL5, which can have a protective effect on disease progression by HIV [105,106]. Lymphocytes T CD8+ recovered from patients infected with HTLV-2 spontaneously produce high levels of chemokines [107] and these chemokine molecules are natural ligands for the CCR5, the most important co-receptor for input of HIV in cells, suppressing the infection with HIV strains with tropism for macrophages [108,109]. Thus, an association between increased production of chemokine and slower disease progression by HIV [110,111], and between increased production of chemokines and reduction of HIV [112] levels has been reported, and slower depletion of CD4+ T cells in individuals with co-infection HIV-1/HTLV-2 [105,113,114,115], as well as lower plasma HIV RNA than for the mono HIV-infected subjects [116].

## 7. HCV and Genetic Susceptibility

Approximately 20%–30% of individuals infected with HCV will clear the virus spontaneously [117], and around of 185 million are infected with this virus worldwide [118]. Chronic hepatitis C is the major cause of hepatocellular carcinoma, end-stage liver disease, and liver transplantation in the USA [117]. Genome-wide association studies (GWAs) identified variants located in the interferon lambda region, and it is associated with HCV clearance spontaneously in subjects under treatment with interferon alfa [26], and interferon lambda 4 (*IFNL4*) plays a fundamental role in these associations [119]. Interferon lambda 4 protein (IFN-λ4) was found in persons who have the *ΔG* allele of the ss469415590 variant (*IFNL4-ΔG*), another important variant is rs12979860, which was associated with spontaneous HCV clearance [120,121] and is a SNP located in IL28B.

Indeed, the linkage disequilibrium is strong among *IFNL4-ΔG* allele and *rs12979860-T* allele is unfavorable in individuals of European or Asian ancestry; nevertheless, this linkage disequilibrium is moderate in individuals of African ancestry [119]. Among black participants, *IFNL4-ΔG* genotype was associated with spontaneous HCV clearance more strongly than *rs12979860* genotype [120,121]. On the other hand, the linkage disequilibrium in the SNP of IL28B in *rs8099917-T* was described as an important predictor of sustained virologic response in chronic HCV subjects undergoing treatment with pegylated alfa interferon and ribavirin over 48 weeks [16].

Other mutations in the innate immune response regulator genes have important roles in the response to treatment in patients with chronic HCV infection, which emphasizes the important role of *KIR* and *HLA* genotypes [40,122]. The variant *KIR2DS3* gene was described as the principal gene associated with chronic HCV infection, whereas the reduction of *HLA-Bw4+ KIR3DS1+* was associated with an increased risk of developing hepatocellular carcinoma. Therefore, they have a role in the innate system in developing HCV-related disorders, of which *KIR2DS3* and *KIR2D* genes stand out as related to HCV disease progression, and lymphoproliferative disorders [122]. This was observed in Brazil, and the most important *KIR* variants associated with SRV was *KIR2DS5*, whereas *KIR2DL2* was associated with chronic hepatitis C [123].

## 8. Concluding Remarks

There are several studies on the immune response against persistent viral infections. In contrast, only a small number of studies on genetic markers have been published recently. This review highlights some specific *HLA* alleles that have been associated with protection or with increased risk of HAM/TSP development; *KIR2DL2* and *IFN-λ3 rs8099917* (GG) polymorphism, associated with HLA class I-restricted, may be involved in the pathogenic mechanism of HIV and HCV infections. All of these polymorphisms should be studied in the future as potential markers in HTLV-1, as well for HIV and HCV infected subjects.
